# A review of the journal achievements since 2017

**DOI:** 10.4102/sajp.v79i1.1983

**Published:** 2023-10-26

**Authors:** Aimee V. Stewart

**Affiliations:** 1 Department of Physiotherapy, School of Therapeutic Sciences, Faculty of Health Sciences, University of the Witwatersrand, Johannesburg, South Africa

I will be stepping down as the Editor-in-Chief of the journal at the end of October 2023 after a fulfilling and eventful period. As such, I felt that a review of the period while I have been Editor-in-Chief of the *South African Journal of Physiotherapy* (SAJP) would be useful. So, what has the team consisting of editors, reviewers, authors, publishers and the South African Society of Physiotherapy (SASP) sponsorship achieved? As always, every achievement is because of teamwork, and the above team has contributed in every way to the increasing success of our journal.

We have a new cover design that was chosen to reflect the caring hands of physiotherapists as well as our diversity. It is also like the SASP logo that we all appreciate.

When I became the Editor-in-Chief, I was advised that we needed to increase the size of our editorial board to comply with international standards of scientific journals and to ensure that we had a better chance of being considered for various indexing services. This was carried out and we now have a board consisting of many South African and international research experts who are interested in supporting and promoting the journal. All board members have a 3-year term after which they are invited to stay for another term. Given that some do not take up the invitation of another term we ensure regular ‘new blood’ on the board.

We also needed to increase the number of articles and this we have done and now regularly publish between 30 and 40 articles annually. This has meant an increase in the SASP subsidy, which has been agreed by the SASP and for which we are grateful. It also has meant hard work for the editorial team and the publishers. As such from 2024, there will be two Associate Editors to assist the Editor-in-Chief.

The reviewer list has been revamped: all reviewers now have a minimum of a master’s degree and two publications. They are divided into the main specialist areas of physiotherapy so that they review in an area in which they have expertise. We regularly send out invitations for new reviewers. We are currently working on a system to enable reviewers to claim Continuous Professional Development (CPD) points, and this will compensate them in a small way. Without our loyal hard-working reviewers, there would be no journal.

We started inviting local physiotherapy experts to write ‘State-of-the-Art’ articles in their field of expertise and have now published a number of these that have been excellent showcases of what their fields contain. I believe that these articles have added to the quality of our journal and showcase what good research academics and clinicians we have.

The journal now has compliance check lists that authors must complete when they are submitting their articles. They must state if they had statistical support and then complete appropriate checklists based on the type of article that they are submitting. These check lists are based on the *CONSORT STATEMENT, STROBE* document, and so on. This has improved the quality of our submissions as all authors can now see what they need to include in their articles. We brought a statistician on board to check the statistics of many of our articles, and this is in line with most journals where articles go through a statistical check prior to being sent out for review.

Our international readership and authorship have increased remarkably as has our local authorship as authors now consider the journal as one of the journals they consider when publishing. To be considered as one of the journals when publishing is an indication of how far the journal has come.

We were listed on PubMed in about 2019, and this immediately made a difference to our visibility. Since then, we have been listed on SCOPUS and have a Cite Score. The best news is that we are now listed on the Web of Science and have an Impact Factor. Having an Impact Factor means a great deal to researchers in health sciences, and this is a huge gain for the journal. In addition, we have now been listed on SciELO, which is also a feather in our collective caps! All this information is on the journal website.

I believe that the SAJP is in a ‘good place’ and I leave feeling satisfied with what we have achieved. It does mean, however, that the hard work must continue. The editors need to work on the journal every week, and we continue to be reliant on the good services provided by our team at AOSIS. The sponsorship of the journal by the SASP is essential as we showcase what our South African profession does. My best wishes to the new editorial team of Prof Witness Mudzi, Editor-in-Chief and the two Associate Editors Dr Annalie Basson and Dr Serela Ramklass.

